# Editorial: Innovators in chemical biology

**DOI:** 10.3389/fchem.2022.1074759

**Published:** 2022-11-04

**Authors:** John D. Wade, Olivier Renaudet, Matthew A. Coleman

**Affiliations:** ^1^ Florey Institute of Neuroscience and Mental Health, Parkville, VIC, Australia; ^2^ School of Chemistry, University of Melbourne, Melbourne, VIC, Australia; ^3^ Universite Grenoble Alpes, Grenoble, France; ^4^ Physical and Life Sciences Directorate, Lawrence Livermore National Laboratory, Livermore, CA, United States; ^5^ Radiation Oncology, University of California, Davis, Sacramento, CA, United States

**Keywords:** amyloid, antimicrobial, biomaterials, GPCRs, natural products, polyketides, peptides, photoreceptors

The science of chemical biology has matured greatly in the past decade to provide enormous insights into the chemical basis of cellular and biological processes and to exploit the resulting knowledge for the development of novel research tools and compounds in across fields such as medicine, agriculture, veterinary science and biology. Such advances have been made possible by not only the acquisition of new chemical and biophysical techniques and instrumentation including cryo-electron microscopy but also superbly skilled application of these.

In this dedicated Research Topic, eleven leading international researchers of chemical biology together with their teams present their latest results across a wide spectrum of research. These showcase the contribution that current or novel chemical biological methods and technology make towards the better understanding of the chemical basis of biology. Application of such expertise and resources is wide and varied as exemplified by the following examples. Avalon et al. report the use of bioinformatic tools to develop predictive models of the biosynthesis of the marine polyketide natural product, palmerolide A. Enzymatic reactions and organic synthesis interpretations were based on homology analyses. Together, these led to the identification of a biosynthetic gene cluster from an Antarctic microbial species that is ultimately responsible for the stepwise synthesis of the polyketide.

The development of new antibiotics is essential given the increasing development of antimicrobial resistance (AMR). Here, Fang et al. focus on the design and synthesis of a series of calix (4) arene derivatives as antimicrobial agents that biomimic the structural properties and biological activities of antimicrobial peptides (Fang et al.). Following the introduction of cationic hydrophilic moieties and after further structural optimization, a lead compound was obtained with potent activity against Gram-positive bacteria while having low toxicity towards mammalian cells. Such rational peptide drug design on novel natural product templates affords a promising path towards addressing AMR.

The reported studies of Wodzanowski et al. were an attempt to better understand the molecular mechanisms whereby the human immune system can differentiate between pathogenic bacteria and the commensal bacteria of the microbiome (Wodzanowski et al.). A comprehensive chemical biology platform was established in which biorthogonal chemistry was used to differentially label the cell walls of two different species of bacteria. Then, a hydrogel-based synthetic matrix was developed and employed to encapsulate monocytes and macrophages for subsequent measurement of the invading bacteria and a reflection of pathogen invasion and homeostatic maintenance.

The fourth manuscript, by Mackinnon et al., in this special Research Topic describes an effort to develop novel inhibitors of hydroxy acid oxidase 1 (HOA1) as an alternative approach to treat primary hyperoxaluria (Mackinnon et al.). They report the use of X-ray crystallography for the discovery and optimization of six low-molecular-weight active site fragments. Two of these were shown by biophysical analyses to be of suitable potency while devoid of substrate competition that make them suitable for further possible drug development.

Lin et al. report their ongoing efforts to develop novel glucose-responsive insulin analogues which represent a primary goal in the better management of fluctuating blood glucose levels in type 1 diabetes and some type 2 diabetes (Lin et al.). Using elegant chemical peptide synthesis, they prepared an insulin analogue that contains two fluorophenylboronic acid (FPBA) moieties at the C-terminus of the A-chain of insulin glargine. In turn, they showed that this led to an increase in the baseline glucose-dependent solubility of insulin without potency reduction. This finding augurs well for the development of novel insulin-based therapies for diabetes. Crystal Chan et al. also use chemical peptide synthesis to prepare a series of analogues of Peptide5, a connexin43 inhibitor, that regulates both cellular communication with the cytoplasm and cell to cell communication (Crystal Chan et al.). The goal was to develop greater *in vivo* stability as well as to increase the peptide’s potency. From these studies, design criteria were established which enabled the production of improved analogues that were suitable for further optimization.

G-coupled receptors are a fascinating and important class of cellular signal transducers which are also primary drug targets for many therapeutic interventions. The review by de Grip and Ganapathy focuses upon rhodopsin proteins, a superfamily of photoreceptors that are essential to multiple elements of light-sensitive animal physiology (de Grip and Ganapathy). Discussions include spectral and structural properties of these proteins together with the current and future applications of engineered rhodopsins in fields as diverse as bioelectronic and biomimic nanotechnology, optogenetics, and cell factories.

Post-translational modifications (PTMs) of peptides and proteins is a key cellular process that adds significant structural and functional diversity. Glycosylation is probably the most common PTM but is highly complex and difficult to study. The manuscript by Zhao et al. review the use of chemoenzymatic synthesis strategies to prepare homogenous complex-type N-glycans for use in the preparation of well-characterized glycopeptides (Zhao et al.). Despite significant gains in synthetic efficiency, much more remains to be done to achieve higher, reliable and reproducible yields of these essential core oligosaccharides.

In another review, Landrieu et al. provide a comprehensive treatise on the putative role of protein aggregation (amyloidosis) in neurological disease or systemic diseases such as type 2 diabetes (Landrieu et al.). The biophysical study of the ultrastructural organization of such aggregates has provided sophisticated and discriminating detail of the molecular features that govern the development and features of fibril (amyloid) formation. The role of chemical biology tools in such studies is highlighted with several examples including of protein engineering methods and biorthogonal chemistry for the introduction of protein chemical modifications. Amyloid-beta oligomers (AβO) are one of the most-studied protein aggregates given its still-unconfirmed role in Alzheimer’s disease onset and development. Hilt et al. report the synthesis of a class of bifunctional stilbenes and of measurements *via* a range of sophisticated chemical biology tools of their ability to modulate the conformational toxicity of the aggregates (Hilt et al.). They show that the ability of these designed small molecules to intervene with neuronal AβO strongly suggests a potential therapeutic application for Alzheimer’s disease.

The final paper in this Research Topic, by Zhou et al., describes the isolation and chemical characterization of six novel bibenzyl small molecules (three pairs of enantiomers), blestrinsD-F, from the tubers of *Bletilla striata*. Use of a range of analytical techniques including 1D/2D NMR led to their structural elucidation (Zhou et al.). One of the compounds showed prominent inhibitory activity against three different Gram-positive bacteria which augurs well for further development as a novel antibiotic.

Together this excellent collection of manuscripts highlights the continuing and powerful role chemical biology makes in our understanding of complex cellular processes. The contributions that the development and utility of novel chemical biology research tools produce are amply illustrated. The work described in the manuscripts also shows the acceleration towards translation to the design and production of research diagnostics and potential medicinal therapeutics and agricultural applications. It is hoped that the reader will enjoy and appreciate this Research Topic.

